# Antioxidative activity of oyster protein hydrolysates Maillard reaction products

**DOI:** 10.1002/fsn3.1605

**Published:** 2020-05-26

**Authors:** Shan He, Yaonan Chen, Charles Brennan, David James Young, Kun Chang, Peter Wadewitz, Qingzhu Zeng, Yang Yuan

**Affiliations:** ^1^ School of Chemistry and Chemical Engineering Guangzhou University Guangzhou China; ^2^ Peats Soil and Garden Supplies Whites Valley SA Australia; ^3^ Institute for Nano Scale and Technology College of Science and Engineering Flinders University Bedford Park SA Australia; ^4^ Centre for Food Research and Innovation Lincoln University Lincoln New Zealand; ^5^ College of Engineering, IT & Environment Charles Darwin University Casuarina NT Australia

**Keywords:** antioxidation, enzymatic hydrolyzation, gas chromatography‐mass spectrometry (GC‐MS), Maillard reaction, oyster meat

## Abstract

A two‐step process of enzymatic hydrolyzation followed by Maillard reaction was used to produce oyster meat hydrolysate Maillard reaction products (MRPs). The flavor of oyster meat hydrolysate MRPs was significantly improved through an optimized orthogonal experimental design. Comparisons between the antioxidative activities of oyster meat hydrolysates and their MRPs were made using lipid peroxidation inhabitation, hydroxyl radical scavenging radical activity, and radical scavenging activity of 2,2 diphenyl‐1‐picrylhydrazyl (DPPH). These methods indicated that an improvement of Maillard reaction on the oyster meat hydrolysates antioxidative activity. Gas chromatography‐mass spectrometry illustrated that the increase was due to the newly formed antioxidative compounds after Maillard reaction, mainly of acids from 22.45% to 37.77% and phenols from 0% to 9.88%.

## Graphical abstract

Optimized process for oyster meat Maillard reaction products with the best sensory. Increased antioxidative activity of oyster meat Maillard reaction products. Increased antioxidative (phenolic and acids) compounds after Maillard reaction.

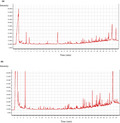

## 1 INTRODUCTION

Oysters, one of the most available and widely distributed marine food resources, had an annual global production of over 4.59 million tonnes in 2016 (Houcke, Medina, Linssen, & Luten, 2016). As well as being rich in protein and minor elements, especially zinc (Rainbow, Liu, & Wang, 2015), oyster's complex seafood flavor is also stronger than other seafood. Using oyster meat as a raw material, many food products with oyster or seafood flavor have been developed around the world, such as oyster sauce, oyster and mushroom crisps (potato chips), and oyster sachets for instant noodles. Nowadays, value‐adding on seafood products has been highlighted as one of the high priority areas for development within the global seafood industry. Regarding oyster, attention has focused on product development with enhanced oyster flavor by using oyster meat as the raw material.

Various reactions have been reported to enhance the flavor of protein‐based products; among them, enzymatic hydrolyzation and Maillard reaction have shown the most promising results. For instance, Yu and Tan (1990) incorporated 10% spray‐dried protein hydrolysates derived from *Oreochromis mossambicus*, a freshwater fish, into a cracker formulation. Using sensory evaluation, Yu and Tan (1990) found that there are highest scores in the categories of color, crispiness, appearance, and crackers with hydrolysates, respectively. Zhang et al. (2013) found that a cooking temperature of 85°C resulted in a high sensory quality regarding color, flavor, aroma, and soup pattern for a crucian carp soup. The authors reported that soup cooked at 85°C resulted in the highest content of protein hydrolysates. In addition to hydrolyzation, Maillard reactions, caused by sugar and amino groups, can create various compound classes that are associated with different flavors. For example, pyrazine is associated with cooked and roasted flavor, alkyl pyridines are associated with bitter and astringent flavors, and thiophenes are associated with meaty flavor. Foods that typically contain each of these compound classes are heated foods in general, coffee, and heated meat, respectively. Boekel (2006) and Asikin et al. (2014) reported increased nutty and roasted flavor (from 26.52% to 38.59%) of brown cane sugar after one year, which occurred because of the formation of volatile products during Maillard reaction. Xinru, Mengchen, and Huanlu (2018) found an enhanced umami and kokumi taste‐active components in bovine bone marrow extract produced during enzymatic hydrolysis. However, the combined impact of Maillard reaction and hydrolyzation on protein‐based products has not been studied, especially for oyster meat.

Further research using chickpea seed protein showed better antioxidative activity after hydrolyzation to protein hydrolysates by pepsin. The similar enzymatic hydrolyzation also produced protein hydrolysates with stronger antioxidative activity from rainbow trout (Mahsa, Parastoo, & Bahare, 2018), Rapana venosa meat (Fenglei, Ronge, & Xueqin, 2018), and crab shell (Wei, Shiwei, & Shijie, 2017). Some of these antioxidative hydrolysates have been purified and sequenced. Most of these protein hydrolysates contain the antioxidant amino acid histidine (Fuentes, Contreras, Recio, Alaiz, & Vioque, 2015). Research by Farvin et al. (2016) illustrated that increased antioxidative activity of cod protein could be achieved via enzymatic hydrolyzation and found that both free amino acids and low molecular weight positively contributed to the antioxidative activity of protein hydrolysates. Thanasak and Soottawat (2018) reported that increased antioxidative activity of collagen from salmon could be achieved by hydrolyzation assisted by ultrasound. However, as far as is known, the Maillard reaction effect on protein hydrolysates, especially oyster meat hydrolysates, has not yet been conducted. And component profile change of oyster meat hydrolysates before and after Maillard reaction, which is the root cause of Maillard reaction effect, has also yet been conduct.

The objective of this study was to comprehensively understand the effect of Maillard reaction on oyster meat hydrolysates from all‐round perspectives, including enzyme selection, Maillard processing, enhanced oyster flavor by sensory evaluation, antioxidation, and the root cause of antioxidation change before and after Maillard reaction. The outcome of this study not only provides novel in‐depth scientific understanding, but also empowers oyster processing industry to achieve higher profits by value‐adding oyster meat, using advanced process presented in this study.

## 2 MATERIALS AND METHODS

### 2.1 Materials

Fresh oyster material was purchased from a regional seafood market (Huashan, Huadu, Guangzhou, Guangdong, China). The food‐grade enzymes, Neutrase (0.8 U/g), Flavourzyme (3 U/g), and Alcalase (5 U/g), were purchased from Biopharma Co., Ltd. The rest chemicals were sourced from Sigma‐Aldrich Corporation.

### 2.2 Preparation of oyster meat mince

The fresh oyster meat was taken out from the oyster shell and minced thoroughly with a manual mincer. The time between purchase and mincing was within 8 hr. The minced fresh oyster meat was mixed with cryoprotectants (sugar (4 g), sodium tri‐polyphosphate (0.3 g), and sorbitol (4 g) per 500 g minced oyster meat), then packed in plastic bags (100 g per bag), and frozen in −23°C freezer until use.

### 2.3 Selection of the optimal enzyme

The frozen oyster meat mince was defrosted for 30 min in vacuumed plastic bags under running tap water. Twenty grams of defrosted oyster meat mince was mixed with 60 g of tap water and then homogenized using a homogenizer (T25 digital ULTRA‐TURRAX, IKA‐works, Inc). The optimal pH of each enzyme was adjusted (Flavourzyme: 6.5; Neutrase: 6.5; and Alcalase: 8.5). Following pH adjustment, each enzyme was added to a mince‐water mixture with the enzyme/substrate (E/S) ratio of 1.0%. The weight of oyster meat mince was considered as the substrate. The processing temperature of each enzyme was set to its optimal temperature as indicated by the suppliers (Flavourzyme: 55°C; Neutrase: 55°C; and Alcalase: 60°C), and the processing time was set as one hour. The enzyme was deactivated in the water bath (90°C, 20 min) after hydrolyzation. The hydrolyzed liquid was continuously centrifuged at 9,000 *g* for a period of 20 min. The top liquid layer after centrifugation was collected for further processing. Volumes of supernatants were recorded for the following calculation of the degree of hydrolysis (DH).

### 2.4 Determination of DH

The determination of DH was conducted using the method from Halim and Sarbon (2016) with slight modification. A total of 20 ml of the sample mentioned above were collected, volume‐measured, and recorded. 10% TCA soluble material was achieved by adding the supernatant to 20 ml of 20% (*w/v*) TCA. The mixture was rested for a period of 30 min to allow for precipitation, followed by being centrifuged at 9,000 *g* for 15 min. The nitrogen content of the initial 20 g of oyster meat mince was measured using the Kjeldahl method (Gao, Li, Zan, Yue, & Shi, 2015). The DH was calculated by using the formula as follows:

$$\text{DH}\;\left(\%\right)=\frac{\text{Nitrogen content in collected, volume - measured and recorded solution}}{\text{Nitrogen content in 20g starting material of oyster meat mince}}\times 100$$

### 2.5 Processing optimization of enzymatic hydrolyzation using the optimal enzyme

Processing time, the ratio of enzyme to the substrate (E/S), and the ratio of oyster meat mince to water were identified as the three critical factors for hydrolyzation of the optimal enzyme. Each factor was analyzed at three levels, as shown in Table 1. The orthogonal design method was used in the experiments. The DH value of the solution after enzymatic hydrolyzation for each test (Table 1) was evaluated. The hydrolysates solution which was produced was subsequently freeze‐dried to powder, sealed, and kept at 4°C until further use.

**TABLE 1:** Levels of key factors in the production of oyster meat hydrolysate using an orthogonal test

Factor	Level		
	1	2	3
Enzyme/Substrate (%)	2.0	2.5	3.0
Oyster meat mince/water (*w/v*)	1/3	1/4	1/5
Processing time (hr)	2.5	3.0	3.5

### 2.6 Processing optimization of oyster meat hydrolysate maillard reaction products

The freeze‐dried powder produced from the hydrolyzation conditions with the optimal enzyme was used as the initial material for the production of oyster meat hydrolysate MRPs. This powder (10 g) was dissolved in 100 ml tap water in the ratio of 1:10 (*w/v*). Glucose was added to the prepared Maillard reactions solution. With three levels of each factor, time, pH, the starting concentration, and a processing temperature of glucose were identified as the four critical the Maillard reactions factors (Table 2). The experiments were also carried out following orthogonal design. The sensory tests were used to measure the quality of oyster meat hydrolysate MRPs solution for each trial listed in Table 2. The starting material of the oyster hydrolysate solution (10% *w/v*) and the oyster meat hydrolysate MRPs solution produced were freeze‐dried to powder after the optimized processing. Then continuously the powder will be sealed and then kept at 4°C until further use.

**TABLE 2:** Levels of key factors in the production of oyster meat hydrolysate Mallard reaction products (MRPs)

Factor	Level		
	1	2	3
Temperature (°C)	105	115	121
Processing time (hr)	25	30	35
pH	6	7	8
Initial glucose (%; *w/v*)	1.5	2.0	2.5

### 2.7 Sensory evaluation

The evaluation of sensory was conducted in the way described by Shahidi and Kiritsakis (2017) with minor modifications. Fifty recruited volunteers from students and staff in the Guangzhou University (Guangzhou, Guangdong, China) were used in the analysis of flavor characteristics based on the procedures agreed by the university's Human Ethics Research Committee. The sensory panelists were not allergic to seafood products. The private booths which are equipped with software of computerized sensory (Sensory Integrated Management System), and hardware of Boekel (2006) was used for the sensory evaluation. One‐hour discrimination testing training, as well as consecutive five rounds of practice triangle testing, were applied to each volunteer. During the five times of practice tests, volunteers were removed from the panelists' pool if they scored poorly for more than twice. Finally, a total of 30 panelists passed the practice triangle test. Three digital numbers were used to code nine different samples. Each panelist was given an oyster meat hydrolysate MRP sample (50 ml). To minimize the color difference effect between samples, samples were provided in dark black colored containers, and red lights were also involved in the testing booths. Room temperature water was used to wash the palate among samples. According to the four criteria shown in Table 3, all panelists were requested to evaluate the samples' quality. The panelists judged each criterion using a score ranged from 0 to 10 (7.6–10.0 = like very much; 5.1–7.5 = like slightly; 2.6–5.0 = dislike slightly; and 0–2.5 = dislike very much). Evaluations were done by the recorded average numbers of the scores, and there are 40 points as the full score for each trial. The starting above solution before the processing optimization of oyster meat hydrolysate MRPs, which was 10 g freeze‐dried powder produced from optimized hydrolyzation conditions, determined using the optimal enzyme, was dissolved in 100 ml tap water, and was served as control.

**TABLE 3:** Sensory evaluation of peanut meal hydrolysate MRPs

Criteria	Evaluation			
	Like very much (7.6–10.0)	Like slightly (5.1–7.5)	Dislike slightly (2.6–5.0)	Dislike very much (0–2.5)
Transparency	Clear and transparent, no sediment	Translucent, no sediment	Opaque, with sediment	Smeary, with more sediments
Color	Constant, light brown (fawn)	Constant, brown	Not constant, hazel	Not constant, dark brown
Aroma	Pleasant seafood flavor, no burning smell	Light seafood flavor, no burning smell	No seafood flavor, slight fermentation smell	No seafood flavor, strong fermentation smell
Taste	Mild savory and slight caramelized flavor, delightful long finish, no bitterness and no astringency	Strong or light savory, inappreciable caramelized flavor, long finish, no bitterness and no astringency	Salty but not savory, short finish, unpleasant astringency and bitterness	Unacceptably salty, not savory, strong astringency and unacceptably bitter

### 2.8 Amino Acid (AA) composition analysis

The method developed by Lorenzo et al. (2017) was used to determine the samples' amino acid compositions. The freeze‐dried samples in Section 2.4. were hydrolyzed in 6 M HCl (100°C for 25 hr. An ultra performance liquid chromatography (UPLC) 90 system with the high‐resolution RP‐HPLC column was used to quantify their amino acid composition, which has an ACQUITY UPLC system with an ultraviolet (UV) detector from Waters Corporation. For all analyses, a column from Waters AccQ‐Tag Ultra (BEH C18, 2.1 × 100 mm, 1.7 μm, Waters Corporation) was utilized with the 55°C column temperature, detecting at 260 nm wavelength, and a 0.7 ml/min solution flow rate.

### 2.9 Measurement of antioxidative activity

The methods of lipid peroxidation, and radical scavenging activity of DPPH and hydroxyl were applied.

#### 2.9.1 DPPH radical scavenging activity method

Measurements of the DPPH radical scavenging activity were done according to the method described in Xie and Schaich (2014). The two freeze‐dried samples mentioned in Section 2.4. were dissolved in distilled water with concentrations of 0.5, 1.0, 1.5, 2.0, 2.5, 3.0, 3.5, 4.0, 4.5, 5.0, and 5.5 mg/ml. 0.2 ml 0.4 mM DPPH solution in ethanol and 2 ml DI water were mixed and served as a control (*A*_ontrol_) and incubated in the dark environment at 37°C for 40 min. Ethanol (*A*_blank_) was used to replace the DPPH solution as a blank sample. UV‐1600 spectrophotometer was used to measure the sample absorbance (*A*_sample_) at 517 nm after incubation. If the value of *A*_sample_ was low, the DPPH scavenging (DPPH_scav_) activity percentage should be high, which shows the scavenging ability of DPPH will be stronger and is calculated as follows:

$${\text{DPPH}}_{\text{scav}}\left(\%\right)=\left(1-\frac{A_{\text{sample}}-A_{\text{sample control}}}{A_{\text{blank}}}\right)\times 100$$

#### 2.9.2 Hydroxyl radical scavenging activity method

The method from Herraiz and Galisteo (2015) with a minor modification was used to measure the samples' hydroxyl radical scavenging activity. In brief, the two aforementioned freeze‐dried samples in Section 2.4. with different concentrations (1, 2, 3, 4, 5, 6, 7, 8, 9, and 10 mg/ml) were made in DI water. Each sample solution with the volume of 1 100 μl was consecutively mixed with 250 μl phosphate buffer solution (100 mM, pH 7.4), 25 μl ethylenediaminetetraacetic acid (EDTA) solution (10 mM), 25 μL ferrous sulfate solution (10 mM), and 25 μl α‐deoxyribose solution (10 mM). Then, 50 μl hydrogen peroxide solution (10 mM) was added to the mixture, followed by shaking for 30 s and allowed to remain at 37°C for 15 min. Finally, 250 μl trichloroacetic acid (2.8%) and 250 μl tert‐butyl alcohol (TBA) solution (1%) were added to the mixture and mixed thoroughly. Then, the absorbance was measured at 325 nm (*A*_sample_). The blank (*A*_blank_) was served by DI water. Ferrous sulfate solutions were used straightway after creating. The lower the *A*_sample_ measurement, the bigger the hydroxyl radical scavenging activity (%), which indicates the stronger hydroxyl radical scavenging activity. The following equation was used to calculate the sc avenging activity (%):

$$\text{Hydroxyl radical scavenging activity (}\%)=\frac{A_{\text{blank}}-A_{\text{sample}}}{A_{\text{blank}}}\times 100$$

#### 2.9.3 Lipid peroxidation inhibition method

The determination of lipid peroxidation inhibition capacity was done according to Yang and Stockwell (2016). The freeze‐dried samples in Section 2.4. were made in DI water with different concentrations of 5, 10, 15, 20, 25, 30, 35, 40, 45, 50, 55, and 60 mg/ml. Prepared solution (200 ml) was mixed with a 5 ml solution consisting of 1.8% hydrochloric acid (*v/v*), 0.37% trichloroacetic acid (*w/v*), and 15% thiobarbituric acid (*w/v*) and 1 g peanut oil. The mixed solution was heated at 90°C for 6 hr in a water bath to promote a pink pigment formation. Afterward, the mixed solution was cooled sharply in an ice bath, centrifuged at 978 *g* for 5 min, and filtrated. At 532 nm, a spectrophotometer was used to measure the absorbance of the filtrate (*A*_sample_). A blank solution (*A*_blank_) was made by replacing the samples with DI water. The lower the *A*_sample_ measurement, the bigger the lipid peroxidation inhibition capacity (%), which indicates the stronger of the lipid peroxidation inhibition ability. The calculation of lipid peroxidation inhibition capacity (%) was done as below:

$$\text{Lipid peroxidation inhibition capacity}\;\left(\%\right)=\frac{A_{\text{blank}}-A_{\text{sample}}}{A_{\text{blank}}}\times 100$$

### 2.10 Gas chromatography‐mass spectrometry analysis

A TRACE DSQ single quadrupole mass spectrometer from Thermo Fisher was used to perform the GC‐MS analyses, based on the way developed by Verslues (2017) with faint modifications. The GC conditions used were as follows: column: ZB‐5MS (Phenomenex), 0.25 µm film thickness, 30 mm × 0.25 mm; carrier gas: helium; split flow: 10 ml/min; linear velocity: injector temperature: 230°C; constant flow rate at 1.3 ml/min; and column temperature program: start temperature (40°C) held for 1 min, after which, increasing to 310°C at 5°C/min. There are MS conditions: ionization: detection: positive ion; electron impact (70 eV); full scan analyses: 10–600 m*/z* at the rate of two scans per second. Volatile metabolites were eluted by using the solvent front in this method, so GC separation of these analyses began with an initial start temperature at 40°C which was maintained for 2 min. The temperature was increased to 80°C at 10°C/min. The temperature was then kept constant at 80°C for 3 min before increasing to 230°C at a rate of 30°C/min.

### 2.11 Data analysis

Triplication was applied in measurements. The format of mean with standard deviation was used to present data and subjected to the least significant difference (LSD) and one‐way variance analysis (ANOVA) using v15 MINITAB Statistical Software. The *F* value at probability (*p* < .05) was used to judge the significance statistically.

## 3 RESULTS AND DISCUSSION

### 3.1 Selection of the optimal enzyme

Table 1 shows the different impact of the three selected enzymes on the degree of hydrolysis (DH) of oyster meat mince under the same processing conditions (processing time: one hour, E/S ratio: 1%). These three enzymes are the proteases that are commonly used for hydrolyzation of proteins from different sources (He, Franco, & Zhang, 2012). DH is defined as the proportion of broken peptide bonds in protein hydrolysates (Ghribi et al., 2015). It has been broadly used to evaluate the efficiency of enzymatic hydrolyzation toward protein. Fallah, Bahram, and Javadian (2015) used the DH value to indicate the impact of Alcalase and Trypsin on the hydrolyzation of processing by‐products derived from sliver carp and found no significant difference between the DH value (%) of Alcalase (4.94 ± 0.15) and Trypsin (4.60 ± 0.38). Jiahui et al. (2018) compared the efficiency of Alcalase, Pepsin, and Papain on hydrolyzation of soy protein hydrolysates and illustrated that Alcalase yielded the highest DH value of all the enzymes. Table 4 shows that Neutrase provided the highest DH (21.72%) among the three enzymes. Furthermore, Neutrase is also the least expensive (Flavourzyme: US$ 53.50/kg; Neutrase: US$ 31.25/kg; Alcalase: US$ 44.63/kg). Hence, Neutrase was selected as the optimal enzymes for the following study.

**TABLE 4:** Degree of hydrolysis (DH) of oyster meat mince hydrolyzed by Flavourzyme, Neutrase, and Alcalase, under the same processing condition (processing time: one hour, enzyme to substrate (E/S) ratio: 1%)

Enzyme	DH (%)*
Flavourzyme	6.84 ± 0.68^a^
Neutrase	21.72 ± 1.32^b^
Alcalase	8.66 ± 0.72^c^

Note

Among each trial, different superscripts in the same column indicate a significant difference (*p* < .05) according to one‐way ANOVA and least squares difference (LSD) test.

* Average of three readings per trial ± standard deviation.

### 3.2 Optimization of enzymatic hydrolyzation

Table 5 shows the DH value of the oyster meat hydrolysate after each trial based on the orthogonal design. Higher DH value not only indicates higher protein recovery (Ghribi et al., 2015) but also smaller molecular weights of produced protein hydrolysates (Carvalho, Bilck, Yamashita, & Mali, 2018). Fallah et al. (2015) found that, in comparison with the silver carp protein hydrolysates produced from Trypsin (DH value of 4.6%, protein recovery of 26.49%), the higher DH value of Alcalase (DH value of 4.94%) led to higher protein recovery (37.01%). Meinlschmidt, Sussmann, Schweiggert‐Weisz, and Eisner (2016) applied five enzymes (Alcalase, Pepsin, Papain, Corolase, and Flavourzyme) to hydrolyze soy protein. They found that the increase in DH value of each enzyme resulted in a decrease in the molecular weight range of protein hydrolysates. For example, with the increase in DH value of Alcalase from 2.0% to 13.0%, the molecular weight range of soy protein hydrolysates narrowed from 10–37 kDa to 10–20 kDa. Smaller molecular weights of protein hydrolysates lead to more exposure of amino acids, including the amino acids that are responsible for the flavor of food products. Therefore, the process also enhanced the flavor value. Laohakunjit, Selamassakul, and Kerdchoechuen (2014) reported that the seaweed protein hydrolysates with the highest DH value of 62.91% presented the strongest seafood‐like flavor. They also reported the highest DH value resulted in the smallest molecular weights of seaweed protein hydrolysates, which led to the greatest exposure of amino acids that possessed seafood flavors, such as glutamate, aspartic acid, and asparagine. Hence, due to the positive reflection of protein recovery and flavor enhancement, we selected the DH value to evaluate the effectiveness of enzymatic hydrolyzation by the optimal enzyme.

**TABLE 5:** Orthogonal design experiment results and analysis of processing optimization for Neutrase

Trial	Factors			
	Enzyme/substrate (^%^) (A)	Oyster meat mince/water (*w/v*) (B)	Processing time (*hours*) (C)	DH (%)*
1	1	1	1	24.16^a^ ± 1.03
2	1	2	2	26.0^a^ ± 0.96
3	1	3	3	22.28^b^ ± 0.82
4	2	1	2	21.28^c^ ± 0.53
5	2	2	3	24.62^a^ ± 1.32
6	2	3	1	25.52^a^ ± 0.78
7	3	1	3	25.35^a^ ± 1.23
8	3	2	1	22.07^b^ ± 0.63
9	3	3	2	24.09^c^ ± 0.85
*K*_1_ ^†^	73.15	72.05	73.57	
*K*_2_ ^†^	71.98	72.70	76.88	
*K*_3_ ^†^	71.51	71.89	66.19	
*R* ^‡^	0.54	0.27	3.57	
The impact of factors	C > A > B			
Optimized processing condition	A_1_B_2_C_2_			

Note

Among each trial, different superscripts in the same column indicate a significant difference (*p* < .05) according to one‐way ANOVA and LSD test.

* Average of three readings per trial ± standard deviation.

^†^ *K*_1,_ *K*_2,_ and *K*_3_ indicate the sum of the DH values corresponding to level 1, level 2, and level 3, respectively.

^‡^ *R* = Max *K*_i_ – Min *K*_i_ (*I* = 1, 2, or 3).

The correlation coefficient (*R*) value in Table 5 indicates the importance of the factor. *R*‐value demonstrates that the processing time was the factor with the most impact (*R* = 3.57). Higher *K* values in each column show stronger impacts. Therefore, evaluated by *K* values alone, the optimized processing conditions were A_1_B_2_C_1,_ which represented a processing time of 3 hr, an enzyme/substrate ratio of 2.0%, and a ratio of oyster meat mince/water of 1:4. The oyster meat hydrolysates produced from this optimized processing condition were used for the studies below.

### 3.3 Processing optimization oyster meat hydrolysate MRPs

The oyster meat hydrolysates derived from the optimized conditions using the optimal enzyme Neutrase was applied as raw material. Table 6 shows the sensory score of oyster meat hydrolysate MRPs after the completion of each trial. The score of the starting material of oyster hydrolysates solution before Maillard reaction served as control. Table 6 shows that the sensory scores of all oyster meat hydrolysate Maillard reaction products (from 24.8 to 33.8) were higher than the that of control (22.3). The optimized processing conditions were A_2_B_1_C_3_D_1_, which represented 115°C in processing temperature, 35 min in processing time, an initial pH of 7, and 2% (*w/v*) of initial glucose concentration. Under this optimized processing condition, the oyster meat hydrolysate MRPs was produced from and applied to the following studies.

**TABLE 6:** Orthogonal design experiment results and analysis of processing optimization for oyster meat hydrolysate Mallard reaction products (MRPs) production

Trial	Factor				Sensory score
	Temperature (°C) (A)	Time (min) (B)	pH (C)	Initial glucose (%) (D)	
1	1	1	1	1	24.8
2	1	2	2	2	28.4
3	1	3	3	3	28.2
4	2	1	2	3	32.4
5	2	2	3	1	33.2
6	2	3	1	2	31.8
7	3	1	3	2	31.4
8	3	2	1	3	28.2
9	3	3	2	1	33.8
*K*_1_ *	81.4	88.6	84.8	91.8	
*K*_2_ *	97.4	89.8	94.6	92.0	
*K*_3_ *	93.4	93.8	92.8	88.8	
*R* ^†^	16	5.2	9.8	3.2	
The impact of factors	A > C > B > D				
Optimized processing condition	A_2_B_3_C_2_D_2_				
Oyster hydrolysates produced from optimized condition by optimal enzyme (control)					22.3

* *K*_1,_ *K*_2,_ and *K*_3_ indicate the sum of the sensory scores corresponding to level 1, level 2, and level 3, respectively.

^†^ *R* = Max *K*_i_ – Min *K*_i_ (*I* = 1, 2, or 3).

### 3.4 Amino acid compositions of oyster meat hydrolysates and oyster meat hydrolysate MRPs

The amino acid oyster hydrolysates compositions produced from optimized processing conditions and oyster meat hydrolysate MRPs generated from the optimized processing conditions are illustrated in Table 7. The oyster meat hydrolysates' total amino acid content reduced from 819.37 mg to 636.60 mg after the Maillard reaction. Maillard reaction happens between reducing sugars and amino acids. This reaction gives food distinctive flavors. It is caused by carbonyl groups of the reducing sugar and amino acids of the protein hydrolysates (Richarme, Marguet, Forterre, Ishino, & Ishino, 2016). The required amino acids during Maillard reaction resulted in the decrease of the total amino acids contents after the Maillard reaction. Among all the amino acids, the three amino acids that reduced the most after Maillard reaction were His (from 72.17 to 35.32 mg/g), Gly (from 88.11 to 50.36 mg/g), and Pro (from 69.62 to 45.96 mg/g). It has been reported that these three amino acids react more frequently than others during Maillard reactions if glucose was applied. Tanaka, Chiu, Nagashima, and Taguchi (1988) reported that histamine reacted with glucose the most among all amino acids during Maillard reaction of sardine protein hydrolysates, which led to an increase in the sardine protein hydrolysates antioxidative activity. Due to the importance of Gly reacted with glucose during the Maillard reaction, the kinetic model of Gly/Glu Maillard reaction pathways were comprehensively studied by Martins and Boekel (2005). They found the intermediate products and end products included *N*‐(1‐deoxy‐d‐fructos‐1‐yl)‐glycine, 3‐deoxy‐2‐hexosulose and 1‐deoxy‐2,3‐hexodiulose, methylglyoxal, and 5‐hydroxymethylfurfural (HMF). Oh, Hartman, and Ho (1992) reported the extensive reaction of Pro and Gly with glucose during Maillard reaction generated pyrrolizine and pyridine volatile compounds, which are responsible for the flavor of food systems.

**TABLE 7:** The amino acid composition* of oyster hydrolysates produced from optimized processing conditions and oyster meat hydrolysate MRPs produced from the optimized processing conditions

Amino acid	Oyster hydrolysates produced from optimized processing condition (mg/g)	Oyster meat hydrolysate MRPs produced from the optimized processing condition (mg/g)
Thr	8.18^a^ ± 0.2	6.62^b^ ± 0.1
Val	10.45^a^ ± 0.1	8.84^b^ ± 0.2
Met	35.38^a^ ± 0.3	30.79^b^ ± 0.3
Ile	13.44^a^ ± 0.1	11.64^a^ ± 0.3
Leu	23.30^a^ ± 0.1	20.13^b^ ± 0.3
Phe	4.72^a^ ± 0.2	4.07^b^ ± 0.2
Trp	100.8^a^ ± 0.1	86.69^b^ ± 0.6
Lys	47.52^a^ ± 0.3	39.86^a^ ± 0.4
His	72.17^a^ ± 0.4	35.32^b^ ± 0.6
Arg	8.52^a^ ± 0.2	7.24^b^ ± 0.2
Asp	50.73^a^ ± 0.3	43.95^b^ ± 0.2
Ser	10.02^a^ ± 0.1	9.03^b^ ± 0.3
Asn	29.50^a^ ± 0.1	25.72^b^ ± 0.1
Glu	101.78^a^ ± 0.3	85.45^b^ ± 0.1
Gln	10.95^a^ ± 0.2	9.54^b^ ± 0.3
Tyr	17.07^a^ ± 0.2	15.24^a^ ± 0.1
Pro	69.62^a^ ± 0.1	45.96^b^ ± 0.1
Gly	88.11^a^ ± 0.1	50.36^b^ ± 0.2
Ala	7.17^a^ ± 0.2	6.83^b^ ± 0.1
Cys	109.94^a^ ± 0.1	93.32^b^ ± 0.3
Total amino acid content	819.37	636.60

Note

Among each trial, different superscripts in the same row indicate a significant difference (*p* < .05) according to one‐way ANOVA and LSD test.

* Average of three readings per trial ± standard deviation.

### 3.5 Antioxidative activities of oyster meat hydrolysates and oyster meat hydrolysates MRPs

The antioxidative activity has been studied in hydrolysates of various protein‐based products, such as egg yolk (Zambrowicz et al., 2015) and fermented mussel (Chi et al., 2015). For example, Chi et al. (2015) separated the hydrolysates from fermented mussels with the amino acid sequence of FGHPY, and this purified peptide with the concentration of 64.8 μM could cleanse 89.5% of hydroxyl radical in radical scavenging assays, determined by electron spin resonance spectroscopy. This represented strong antioxidative activity. Zambrowicz et al. (2015) produced hydrolysates from egg yolk and showed that hydrolysates permeate antioxidative activity of from a 10 K membrane can surpass the antioxidative activity of alpha‐tocopherol, which is one of the recognized commercial antioxidant.

**FIGURE 1:**
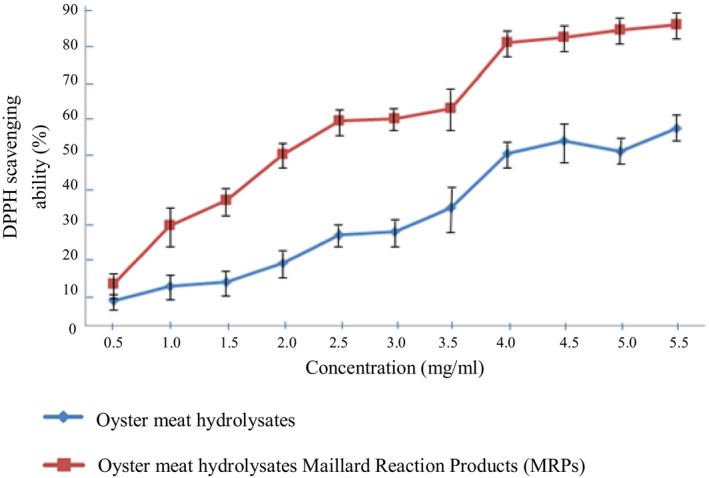
Comparison of DPPH scavenging ability between oyster meat hydrolysates and oyster meat hydrolysate Maillard reaction products (MRPs)

**FIGURE 2:**
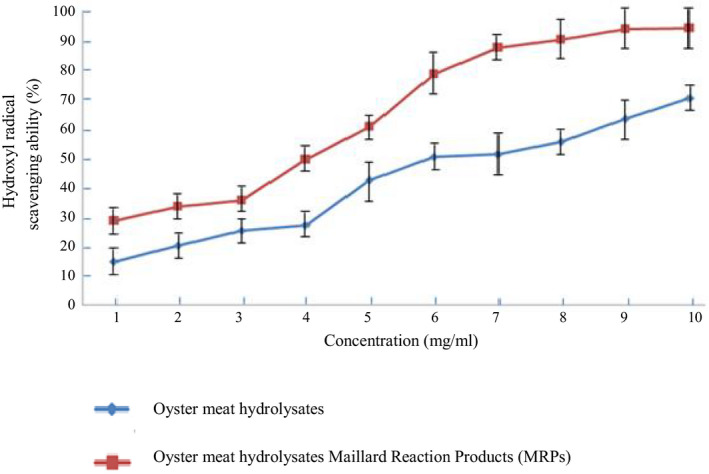
Comparison of hydroxyl radical scavenging ability between oyster meat hydrolysates and oyster meat hydrolysate MRPs

**FIGURE 3:**
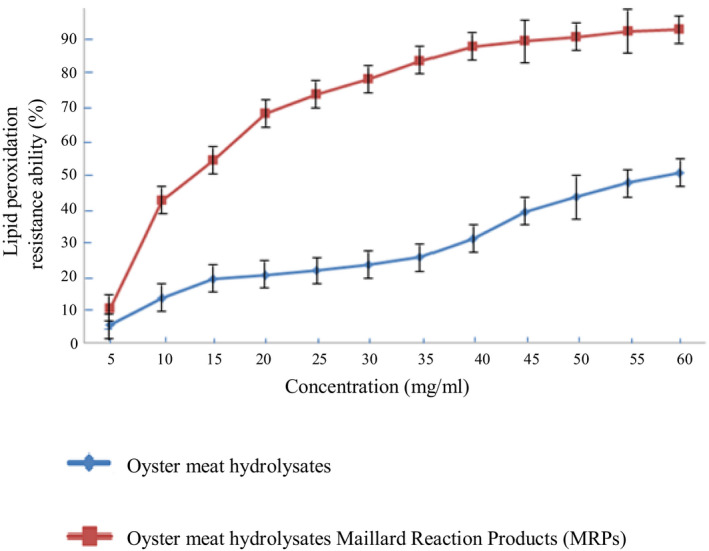
Comparison of lipid peroxidation resistance ability between oyster meat hydrolysates and oyster meat hydrolysate MRPs

By using three methods to measure antioxidative activity, our study reported that the antioxidative activity of oyster meat hydrolysates rose after the Maillard reaction (Figures 1, 2, 3). Furthermore, they also showed that the gap between the antioxidative activity of the oyster meat hydrolysate MRPs and oyster hydrolysates increased with an increase in concentration. For instance, the lipid peroxidation resistance ability of the 5 mg/ml enzymatic hydrolysates showed no significant difference before and after the Maillard reaction. However, at 60 mg/ml concentration, the gap grew to approximately 40%. The results from the measurements of the three methods were in line with each other and therefore strongly confirmed the growth in the antioxidative activity of oyster meat hydrolysates caused by Maillard reactions.

### 3.6 GC‐MS analysis of oyster meat hydrolysates and oyster meat hydrolysate MRPs

**FIGURE 4:**
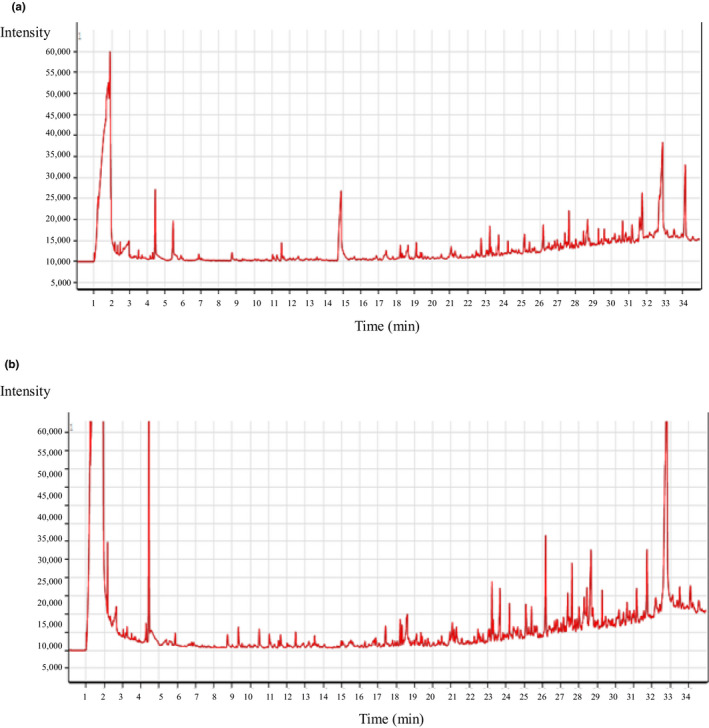
Gas chromatography‐mass spectrometry (GC‐MS) analysis of oyster meat hydrolysates and oyster meat hydrolysate MRPs: (a) oyster meat hydrolysates and (b) oyster meat hydrolysate MRPs

The increase of antioxidative activity of oyster meat hydrolysate MRPs illustrated the possible change regarding the components of oyster hydrolysates affected by Maillard reaction. GC‐MS analysis was applied to investigate the components of oyster hydrolysates and oyster meat hydrolysate MRPs, respectively (Figure 4) to understand the change. Several peaks only appear in the graph of oyster meat hydrolysate MRPs (Figure 4b). This indicates that there is no formation of new components after the Maillard reaction. The components and their contents are shown in the two drawings of Figure 4 were further measured by NIST14.L library retrieval analysis (Table 8). It can be seen from Table 8 that the two group of components that increased the most after Maillard reaction are acids (from 22.45% to 37.77%) and phenols (from 0% to 9.88%). Phenols' antioxidative activity has been frequently reported previously. Vinson, Proch, and Zubik (1999) tested the content of phenol in cocoa, dark chocolate, and milk chocolate, and found the antioxidative activity of these foods is positively correlated with their phenol content. The two phenols appeared after Maillard reaction were maltol and 5‐methyl‐2‐isopropylphenol. Long, Kwee, and Halliwell (2000) identified the antioxidative compounds in dark soy sauce. They identified that maltol was one of several active compounds. Dintcheva, Baiamonte, and Spera (2018) incorporated 5‐methyl‐2‐isopropylphenol in polylactic acid at 2 and 3 wt%, and found its antioxidative activity in water was enhanced. Furthermore, many acids' percentages increasing after Maillard reaction were also positively related to the system responsible for the increase in antioxidative activity. For example, acetic acids increased from 7.97% to 10.97% after the Maillard reaction. Kaya, Akram, and Ashraf (2018) indicated that the spray of acetic acid at 2 mM could mitigate the negative impact on B toxicity in maize plants, which is associated with stronger activities key antioxidant enzymes in maize plants. Fumaric acid increased from 0.72% to 2.46% after the Maillard reaction (Table 8). Wang et al. (2015) found that after the modification of chitosan in an ionic liquid solution by using fumaric, the antioxidative activity increased from 63% to 85%, measured with the DPPH radical scavenging method. Therefore, the components change after the Maillard reaction is the cause of the positive influence of the Maillard reaction on antioxidative activity.

**TABLE 8:** Comparison between the components of oyster hydrolysates and oyster meat hydrolysate MRPs

Category	Compound	Percentage (%)	
		Oyster meat hydrolysates	Oyster meat hydrolysates MRPs
Alcohol	1‐Octen‐3‐ol	9.11	10.21
	Furfuryl alcohol	5.12	3.88
	Phenethyl alcohol	2.15	2.98
	Cyclopentanol	–	2.51
	tert‐Butanol	1.82	3.13
	1‐Penten‐3‐ol	1.51	2.75
	2‐Penten‐1‐ol	1.38	2.69
	2,3‐Butanediol	1.31	1.47
	1‐Octen‐3‐ol	0.79	0.83
	Furfuryl alcohol	23.19	30.45
	Sum	46.38	30.45
Acid	Acetic Acid	7.97	10.97
	Propionic acid	8.68	7.43
	Oleic acid	1.14	2.97
	Myristic acid	0.95	2.66
	Palmitic acid	1.34	2.85
	Fumaric acid	0.72	2.46
	Benzoic Acid	0.24	2.17
	Stearic acid	0.75	2.43
	Phenylacetic acid	0.66	3.83
	Sum	22.45	37.77
Aldehyde	Benzaldehyde	5.78	1.95
	Acetaldehyde	2.48	0.45
	Hexaldehyde	0.52	–
	Heptanal	1.46	–
	Octanal	0.79	–
	Nonanal	–	0.17
	2‐Pentenal, 2‐methyl‐	1.16	0.45
	Vanillic aldehyde	1.13	0.93
	3‐(Methylthio)propionaldehyde	0.56	0.63
	trans‐2‐Hexenal	0.85	–
	Sum	14.73	4.58
Ketone	2‐Nonanone	0.41	0.12
	Acetone	3.51	1.77
	2‐Butanone	0.87	0.14
	1‐Penten‐3‐one	1.06	0.46
	2‐Piperidone	0.23	0.14
	3‐Penten‐2‐one	–	0.35
	2‐Heptanone	0.68	0.37
	Benzylidene acetone	–	0.51
	2,6‐Dimethyl‐4‐pyrone	0.17	0.46
	Sum	6.93	4.32
Hydrocarbon	n‐Dodecane	0.76	0.52
	n‐Tetradecane	1.13	0.54
	n‐Hexadecane	0.92	0.78
	n‐Heptadecane	0.72	0.35
	Eicosane	0.57	–
	Tetracosane	0.48	–
	1‐Octadecene	–	0.45
	Longifolene	–	0.64
	Cinene	0.18	0.85
	Sum	4.76	4.13
Sulfur nitride	Methyl sulfide	2.58	0.93
	2,6‐Dimethylpyrazine	–	0.87
	2‐Methylpyrazine	–	1.12
	2‐Acetylfuran	–	1.31
	2,3‐Benzofuran	–	1.19
	Sum	2.58	5.42
Ester	n‐Butyl butyrate	0.41	–
	Ethyl heptanoate	0.32	0.12
	Diethyl phthalate	–	0.73
	Ethenyl ethanoate	0.74	–
	Sum	1.47	0.85
Phenol	Maltol	–	5.93
	5‐Methyl‐2‐isopropylphenol	–	3.95
	Sum	0	9.88
	Total	99.40	97.60

## 4 CONCLUSIONS

We applied a two‐step process including enzymatic hydrolyzation followed by Maillard reaction to create oyster meat hydrolysate MRPs with improved flavor. According to the orthogonal experimental design, the optimized processing conditions for enzymatic hydrolyzation were as follows: processing time of three hours, enzyme/substrate ratio of 2%, and the ratio of oyster meat mince/water of 1:4, with applying Neutrase. The optimized processing conditions for Maillard reaction were as follows: temperature being at 115°C; initial pH being equal to 7; 35 min processing time; and an initial 2% glucose concentration (*w/v*). All three antioxidative activity measurements convinced the increased antioxidative activity of oyster meat hydrolysates by Maillard reaction. GC‐MS measurement was used to explain the reasons: It was due to the increase of antioxidative compounds, especially acids (from 22.45% to 37.77%) and phenols (from 0% to 9.88%).

## CONFLICT OF INTEREST

Authors declare no conflict of interest in this study.

## STUDIES INVOLVING HUMAN SUBJECTS

Authors declare that this study conforms to the Declaration of Helsinki, USA, and/or European Medicines Agency Guidelines for human subjects.

## STUDIES INVOLVING ANIMAL SUBJECTS

Authors declare that this study does not involve animal or human subjects.

## STUDIES INVOLVING PATIENTS

Authors declare that this study does not involve patients.
